# Transcriptome-wide shift from photosynthesis and energy metabolism upon endogenous fluid protein depletion in young *Nepenthes ampullaria* pitchers

**DOI:** 10.1038/s41598-020-63696-z

**Published:** 2020-04-20

**Authors:** Hoe-Han Goh, Anis Baharin, Faris ‘Imadi Mohd Salleh, Rishiesvari Ravee, Wan Nor Adibah Wan Zakaria, Normah Mohd Noor

**Affiliations:** Institute of Systems Biology, Universiti Kebangsaan Malaysia, 43600 UKM Bangi Selangor, Malaysia

**Keywords:** Plant molecular biology, Plant physiology

## Abstract

Carnivorous pitcher plants produce specialised pitcher organs containing secretory glands, which secrete acidic fluids with hydrolytic enzymes for prey digestion and nutrient absorption. The content of pitcher fluids has been the focus of many fluid protein profiling studies. These studies suggest an evolutionary convergence of a conserved group of similar enzymes in diverse families of pitcher plants. A recent study showed that endogenous proteins were replenished in the pitcher fluid, which indicates a feedback mechanism in protein secretion. This poses an interesting question on the physiological effect of plant protein loss. However, there is no study to date that describes the pitcher response to endogenous protein depletion. To address this gap of knowledge, we previously performed a comparative RNA-sequencing experiment of newly opened pitchers (D0) against pitchers after 3 days of opening (D3C) and pitchers with filtered endogenous proteins (>10 kDa) upon pitcher opening (D3L). *Nepenthes ampullaria* was chosen as a model study species due to their abundance and unique feeding behaviour on leaf litters. The analysis of unigenes with top 1% abundance found protein translation and stress response to be overrepresented in D0, compared to cell wall related, transport, and signalling for D3L. Differentially expressed gene (DEG) analysis identified DEGs with functional enrichment in protein regulation, secondary metabolism, intracellular trafficking, secretion, and vesicular transport. The transcriptomic landscape of the pitcher dramatically shifted towards intracellular transport and defence response at the expense of energy metabolism and photosynthesis upon endogenous protein depletion. This is supported by secretome, transportome, and transcription factor analysis with RT-qPCR validation based on independent samples. This study provides the first glimpse into the molecular responses of pitchers to protein loss with implications to future cost/benefit analysis of carnivorous pitcher plant energetics and resource allocation for adaptation in stochastic environments.

## Introduction

Carnivorous plants are commonly found in habitats deprived of nutrients, especially nitrogen and phosphorus. To qualify as carnivorous, a plant must have at least one adaptation for active attraction, capture, digestion, and clear nutritional benefit from carnivory^1^. The convergent evolution of passive pitfall traps resulted in carnivorous pitcher plants of diverse orders and families, including Nepenthaceae (Caryophyllales), Cephalotaceae (Oxalidales), and *Sarracenia*ceae (Ericales) at distant geographical locations but sharing similar digestive fluid proteins^2–4^. A species-rich monotypic group of tropical pitcher plants from genus *Nepenthes* develop attractive pitchers of diverse shapes and sizes^5^. Recently, two highly conserved leaf developmental regulatory genes, *ASYMMETRIC LEAVES1* (*AS1*) and *REVOLUTA* (*REV*), have been shown to be key in pitcher development from the leaf tips^6^.

A typical *Nepenthes* pitcher constitutes three functional zones^7^. First, the attractive zone comprises the lid with nectars as baits to lure insects and the rim known as peristome often with striking colours. Second, the slippery waxy zone with epicuticular waxes covering the peristome and upper inside surface of pitchers^8^. Third, the digestive zone at the lower inside surface of pitchers with glands secreting acidic fluids and hydrolytic enzymes for the digestion and absorption of nutrients through transporters^9,10^. Insects often accidentally fall into pitchers from the slippery waxy peristome^11^. Within the pitcher, the trapped insects are intoxicated by naphthoquinones^12^ before drowned in the viscous digestive fluid^13,14^.

Most studies on *Nepenthes* pitcher fluid have focused on the elucidation of protein composition with various proteins identified over the past two decades, which are currently accelerated by the high-throughput proteomics informed by transcriptomics approach^15–18^. The most abundant enzymes are proteases^3,19^, including aspartic protease Nepenthesins and prolyl endopeptidase neprosins^15,20^. Many proteins found are known to play roles in plant defence against microbial pathogens, such as thaumatin-like proteins and pathogenesis-related (PR) proteins^21^. The findings that many proteases, chitinases, and glucanases found in the pitcher fluids can also be classified as PR proteins led to the inference that offensive carnivory mechanism evolved from existing plant defensive mechanism against herbivory^22^. This is also supported by the conserved jasmonate signalling of enzyme secretion during prey capture^23,24^.

*Nepenthes ampullaria* Jack is a unique species with unusual growth patterns and pitcher morphology compared to other lowland *Nepenthes* species that typically grow in open sunny habitats. *N. ampullaria* produces dense clusters of uncovered squat-shaped wide-mouthed pitchers with highly flexed vestigial lids^25^. During its evolutionary progression towards detritivory, functionally redundant traits for prey attraction (nectar glands) and capture/retention (waxy zone and lunate cells) are lost, which reflect its adaption to leaf litter trapping on the forest floor under shady closed-canopy heath forests or peat swamps^26^. Pitchers of *N. ampullaria* are long-lived with a feeding period of over 8 months^27^ and adapted for slow and steady ammonium (NH_4_^+^) uptake from the slow breakdown of plant materials by commensal microbes, compared to short-lived (≤ 3 months) pitchers with high turnover and prey capture rates^28^. Pitcher fluids of *N. ampullaria* are less acidic to maintain infauna (mosquito larvae and bacteria) for leaf litter decomposition and nutrient mineralisation^29,30^. Over half of the biomass of dead matter in the pitchers was plant-derived^31^, in which >35% of leaf nitrogen (N) is obtained from fallen leaves containing only 1.2% N^26,32^. The adaptive radiation of N acquisition in *N. ampullaria* through plant materials as well as insect prey by retaining a large slippery peristome in prey capture^33^ and endochitinase activities in the pitcher fluid^34^ explains its wide range of distribution and often co-exists with other insectivorous species^25^.

Botanical carnivory for resource acquisition appears to be a costly lifestyle, which suggests that the energetic costs of producing trapping organs, enzymes, waxes, and other secondary meabolites prevent carnivorous plants from being widespread in the plant kingdom over unrestricted habitats^35,36^. Despite recent studies on the prey-induced secretion of digestive enzymes^23,24^, molecular data on the regulation of endogenous protein secretion and replenishment are still lacking to elucidate the molecular and physiological roots of plant carnivory mechanisms. Recently, we reported that certain proteins were replenished even without prey induction in the pitcher fluids of *Nepenthes* x *ventrata* within three days of pitcher opening^37^, which indicates molecular regulation of protein secretion in the pitchers in response to protein loss. In the current study, we performed detailed downstream transcriptomic analyses on the RNA-sequencing (RNA-seq) data from previously reported experiment^38^ to identify transcriptome-wide pitcher response to endogenous protein loss during early pitcher opening. We also repeated the experiment for RT-qPCR validation and reproducibility. The aim of this study is to generate functional annotations for the first reference transcriptome of *N. ampullaria* pitchers for further molecular studies. By using *N. ampullaria* as a model system to study carnivorous plant protein depletion, we address the following questions:

1. How does the gene expression change during early pitcher opening?
2. How the pitchers transcriptionally respond to the depletion of endogenous proteins in pitcher fluids?
3. How the transcriptions of pitcher secretome and transportome are affected by protein depletion?
4. What are the transcriptional regulatory factors responsive to the changes in pitcher fluid proteins?

## Results

### Molecular composition of *Nepenthes ampullaria* whole pitcher trap

A total of 158,756 unigenes and 202,322 transcripts with 55,788 predicted peptide sequences were obtained from *de novo* assembly of 134,043,179 processed Illumina 125 bp paired-end reads from the previous RNA-seq experiment^38^. There was one less unigene compared to the previous report^38^ after counting correction. The transcript lengths range between 224 bp and 11,748 bp, with N50 length of 1,066 bp. The analysis of 55,788 predicted peptide sequences identified a total of 28,238 (50.6%) complete, 14,017 (25.1%) 5′ partial, and 4,887 (8.8%) 3′ partial sequences, while 8,646 (15.5%) were internal sequences without the start and stop codons.

Functional annotation of unigenes through Trinotate pipeline with BLAST searches found hits against different public databases (Table 1), including nr (35,346, 22.3%), Swiss-Prot (19,053, 12.0%), PFAM (18,377, 11.6%), eggNOG (15,577), and KEGG (25,512, 16.1%). Local BLASTN analysis against Arabidopsis genes found 37,911 (23.9%) hits. Furthermore, predictions of signal peptide (SignalP) and protein transmembrane helices (TmHMM) obtained positive results with 1,512 (1.0%) and 5,329 (3.4%) unigenes, respectively. Combining all, a total of 49,871 (31.4%) of unigenes can be functionally annotated while the rest of 108,885 (68.6%) unigenes remained unannotated, in which majority (86,100, 54.2%) are <500 bp in length. Detailed summary on unigene length distribution, functional annotation, sequence homology against nr database, and WEGO analysis can refer to Supplementary File 1. In total, 34,444 (21.7%) unigenes were annotated with gene ontology (GO) terms through BLAST and Pfam analyses, in which 27,646 unigenes categorised in cellular component (mainly cell, cell part, and organelle), 28,839 unigenes in molecular function (mainly binding and catalytic activity), and 26,926 unigenes in biological process (mainly cellular process and metabolic process) (Fig. S1).

**Table 1:** Summary statistics of transcriptome assembly and functional annotation.

Attribute	Value	%
Total unigene	158,756	
Total transcript	202,322	
Total predicted peptide	55,788	
Range (bp)	224–11,748	
N50 length (bp)	1,066	
**Functional annotation**		
nr (BLASTX)	35,346	22.3
Swiss-Prot (BLASTP)	19,053	12.0
Pfam	18,377	11.6
eggNOG (KOG)	15,577	9.8
KEGG (KO)	25,512	16.1
AGI (BLASTN)	37,911	23.9
SignalP	1,512	1.0
TmHMM	5,329	3.4
Annotated	49,871	31.4
Unannotated	108,885	68.6

**Figure 1:**
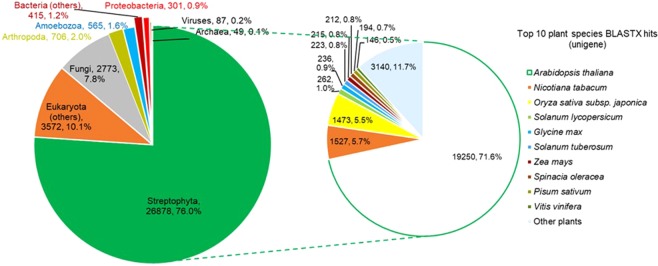
Taxonomic distribution of *Nepenthes ampullaria* unigenes based on BLASTX analysis against the NCBI nr database (E-value cutoff of 1e-5).

Based on taxonomic analysis (Fig. 1), the majority (26,878, 76.0%) of unigenes have BLASTX hits to plants (Streptophyta), in which 71.6% hit to *Arabidopsis thaliana*, followed by *Nicotiana tabacum *(5.7%), *Oryza sativa* (5.5%), and *Solanum lycopersicum* (1.0%). The rest of the annotated unigenes hit to other Eukaryota (3,572, 10.1%), apart from fungi (2,773, 7.8%), insects (Arthropoda, 706, 2.0%), and amoeboid protists (Amoebozoa, 565, 1.6%), which could be derived from symbionts or contaminants as sampling was performed in the field. Only 852 (2.4%) unigenes hit to prokaryotes (mainly Proteobacteria), archaea or viruses. We did not exclude any unigene for downstream analyses because these unigenes might be endogenous to *N. ampullaria*, in which 561 (65.8%) of the 852 unigenes hit to prokaryotes can also find hit to Arabidopsis genes (Supplementary File 1).

To assess the completeness of the assembled transcriptome, we performed Benchmarking Universal Single-Copy Orthologs (BUSCO) analysis, which found 91.3% of 1,375 universal single-copy orthologs in the plant benchmark set to be complete and 5.2% fragmented, while only 3.5% were missing in the pitcher tissue (Fig. S2). Based on this reference transcriptome, we aimed to understand molecular regulation associated with *N. ampullaria* pitcher physiology, protein regulation, and molecular transport.

### Unigene abundance analysis

Transcriptome-wide gene expression was estimated based on alignment results for each sample based on RSEM analysis at the unigene level. More than half of the unigenes were found to have a TPM value of 0 in sample D0 (52.3%) and D3C (54.2%) compared to 42.5% in D3L (Table S2). The majority of unigenes (116,055, 73.1%) have mean TPM < 1, in which 92,945 (58.5%) were unannotated (Supplementary File 1). Of the 1,347 unigenes annotated with BUSCO IDs, 1,316 (97.7%) were found to have mean TPM > 1. Therefore, we focused our downstream functional enrichment analyses on 42,701 (26.9%) unigenes with mean TPM > 1, in which 26,761 (62.7%) were functionally annotated. The top 1% (1,588) abundant unigenes in D0 sample accounted for 68.0% of RNA-seq reads compared to 54.4% and 53.2% for D3C and D3L, respectively. This indicates a more distributed gene expression in D3 pitchers with less dominance of highly expressed unigenes.

**Figure 2:**
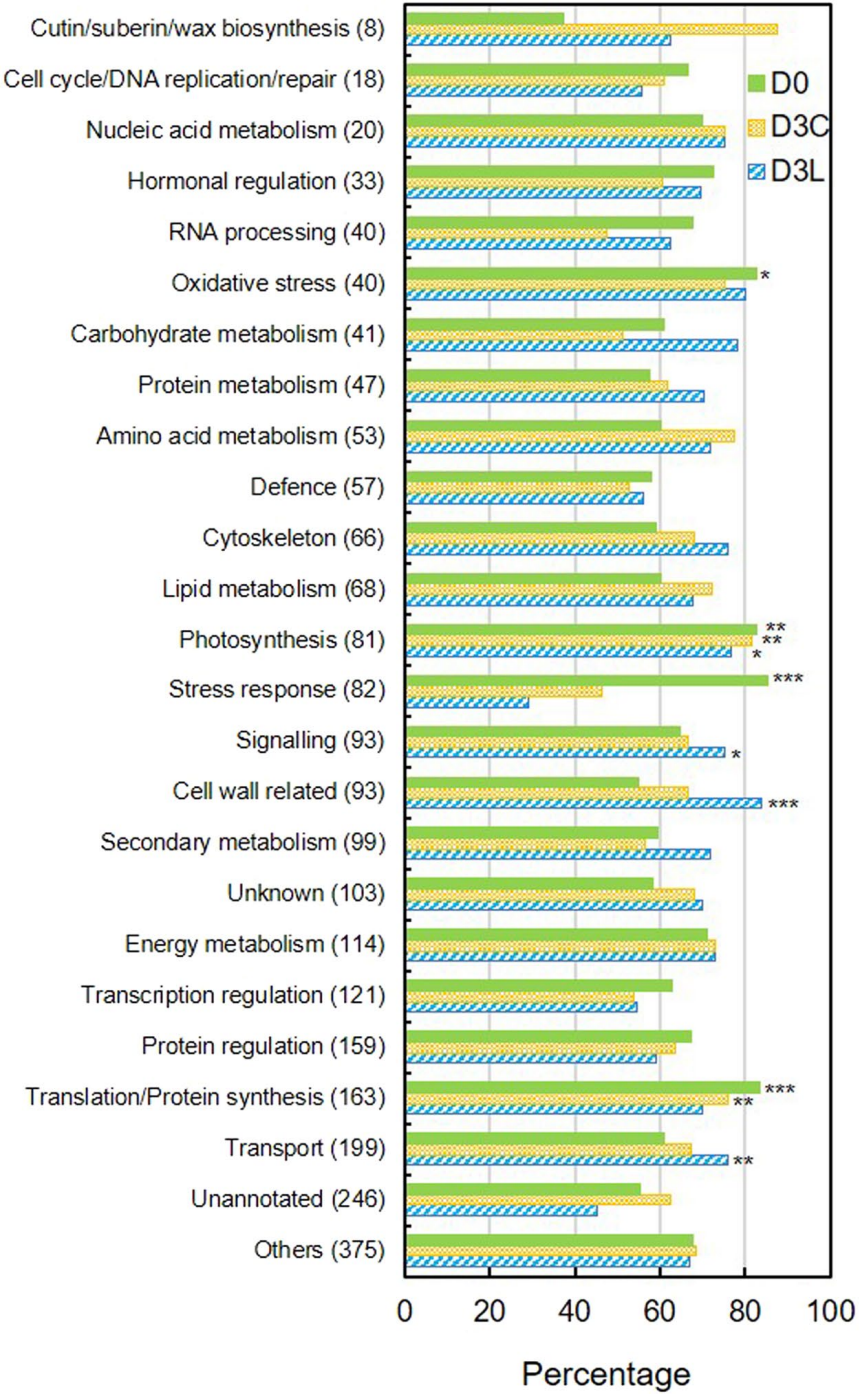
Functional enrichment analysis of the top 1% abundant unigenes based on functional categorisation. The percentage is calculated based on the proportion of the top 1% abundant unigenes in each sample against the total number in all samples (numbers in parentheses) for the respective categories. Fisher's exact test with Benjamini-Hochberg multiple test correction: *FDR < 0.05, **FDR < 0.01, ***FDR < 0.001. Background of 2,419 top 1% unigenes from all three samples. Refer Supplementary File 2 for details.

To investigate the functions of dominant unigenes, we manually categorised the top 1% (1,588) abundant unigenes of each sample into 22 informed functional categories based on UniProt annotations related to main biological processes with reference to the previous literature^15^ in pitcher plants (Supplementary File 2). Unigenes wth known functions not within the 22 categories were classified as “others”, unknown function as “unknown”, whereas those without annotation as “unannotated”. Functional enrichment analysis identified photosynthesis as overrepresented in all three samples based on a background of total 2,419 top 1% unigenes (Fig. 2). Translation/protein synthesis was overrepresented for D0 and D3C but not D3L. Additionally, stress response and oxidative stress were overrepresented in D0 compared to cell wall related, transport, and signalling for D3L.

A Venn analysis was performed on the top 1% abundant unigenes from each sample followed by enrichment analysis of each group of unigenes (Supplementary File 2, Fig. S3). Processes related to photosynthesis, cell wall/membrane biogenesis, translation, protein regulation, water transport, and stress response appeared to be highly enriched based on annotated unigenes in 903 top 1% abundant unigenes common in all three samples. Stress-related heat shock proteins (HSPs) were enriched in 167 top 1% abundant unigenes shared between D0 and D3C compared to carbohydrate metabolism for 185 top 1% abundant unigenes shared between D0 and D3L. Histone was enriched in the top 1% abundant unigenes unique to D0 compared to glucuronoxylan biosynthesis and phytepsin (a vacuolar aspartic endopeptidase) for D3C. Notably, a plant-specific Tify transcription factor (TF) family related to the suppression of jasmonate (JA) response was overrepresented in the top 1% abundant unigenes unique to D3L.

### Differentially expressed gene analysis

**Figure 3:**
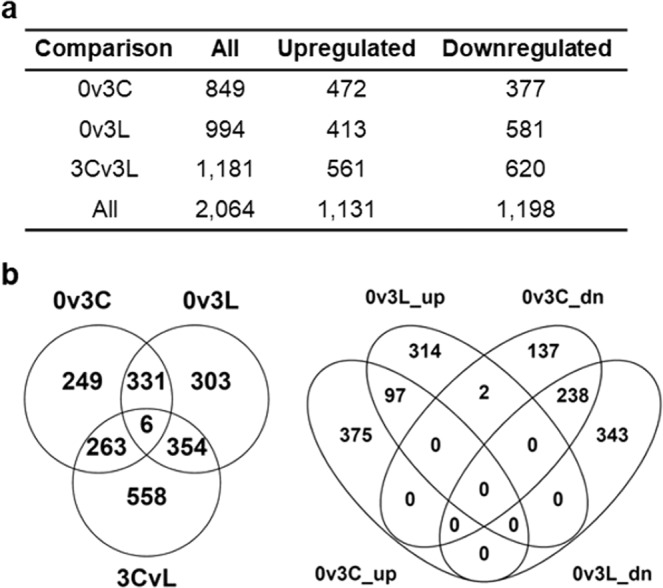
Summary of DEG analysis. (**a**) Statistics of DEGs from different comparisons. (**b**) Venn analyses of DEGs from different comparisons based on overall (2,064), upregulated (up) (1,131) or downregulated (dn) (1,198) DEGs. D0/0: day 0 control; D3C/3C: day 3 longevity experiment; D3L/3L/L: day 3 endogenous protein depletion experiment.

To investigate the transcriptional effect of endogenous protein depletion from the pitcher fluid upon pitcher opening, we identified differentially expressed genes (DEGs) in the three samples using all-versus-all pairwise comparisons (Fig. 3). A total of 2,064 (1.3%) out of 158,756 unigenes were found to be significantly regulated in at least one pairwise comparison with a similar total number of up- (1,131) and down-regulated (1,198) DEGs. The majority (99.7%) of these DEGs have mean TPM > 1 (Supplementary File 1) and 444 (21.5%) DEGs were classified under the top 1% abundant unigenes in at least one sample, especially D0 (Supplementary File 2). DEGs were enriched in processes related to protein regulation, secondary metabolism, intracellular trafficking, secretion, and vesicular transport based on KOG analysis (Fig. S4).

The depletion of endogenous protein from pitcher fluid (0v3L) resulted in the increased number of downregulated unigenes compared with D3 control (0v3C), which was also reflected by a higher number of 3Cv3L downregulated DEGs (Fig. 3a). Venn analyses revealed 337 DEGs to be shared between 0v3C and 0v3L comparisons which represent a common set of unigenes regulated during the 3 days of pitcher opening (Fig. 3b). Of these 337 DEGs, 97 were upregulated and 238 were downregulated. The two DEGs which were 0v3L upregulated but 0v3C downregulated, TR100352|c1_g2 and TR31377|c2_g1, encode a (-)-germacrene D synthase and a disease resistance RPP8-like protein, respectively (Supplementary File 3). On the other hand, 558 DEGs not regulated between D0 and D3 were found significantly regulated in 3Cv3L comparison, which could be the effect of endogenous protein depletion treatment.

**Figure 4:**
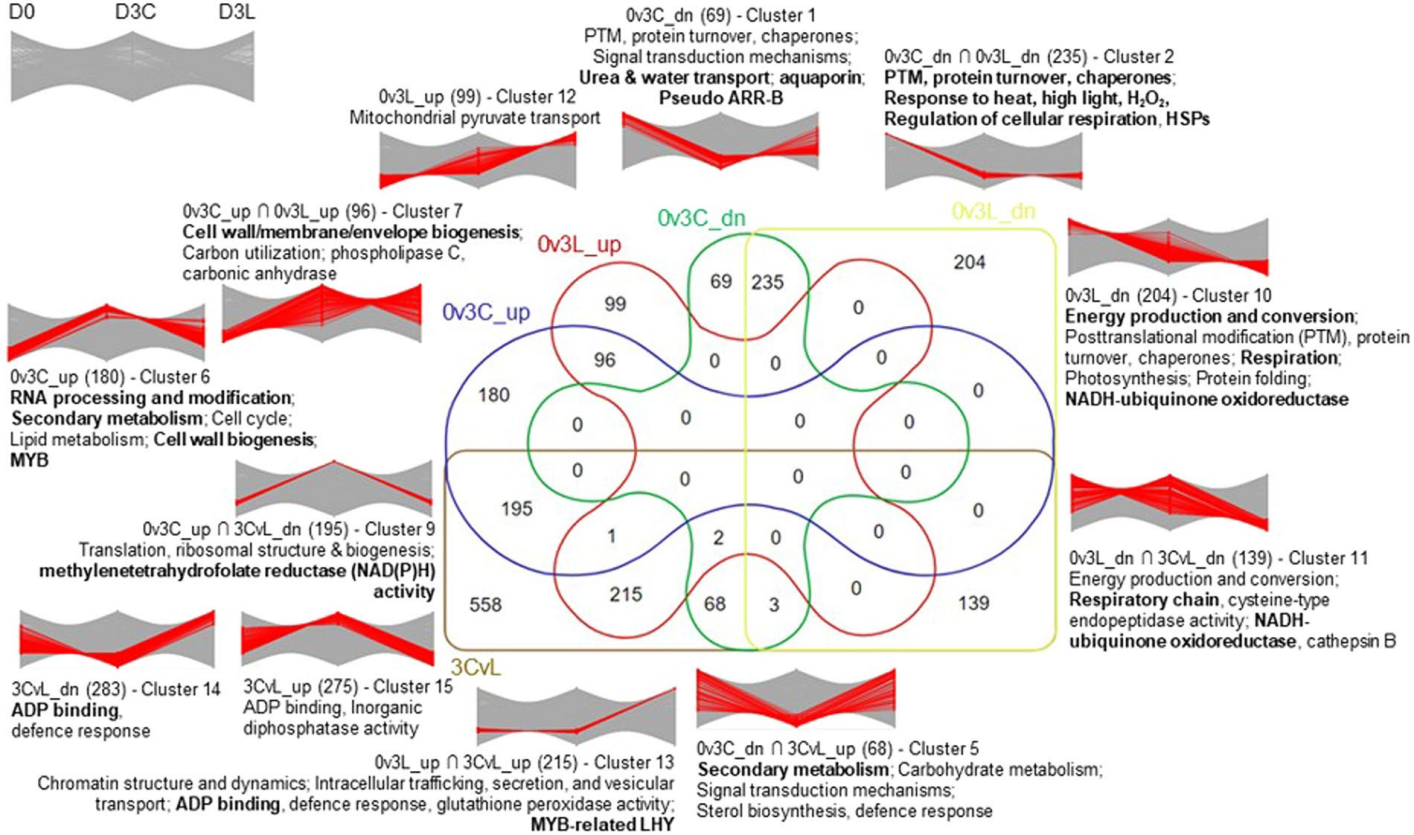
Venn analysis and enrichment analysis of 2,064 DEGs based on all functional annotations. Fisher's exact test with Benjamini-Hochberg multiple test correction cut-off values *FDR* < 0.05. The lists of enriched terms are sorted according to the descending order of enrichment factors based on the background of all unigenes. Bolded fonts represent significant enrichment with a stringent background of only annotate unigenes in the respective categories. Graphs showing different expression patterns of DEGs based on z-score mean-normalised TPM values. Three clusters with less than four DEGs are excluded. Refer Supplementary File 3 for details on the analysis.

To examine the functional significance of these DEGs based on differential expression patterns, a more detailed Venn analysis was performed to include D3CvsD3L followed by enrichment analysis of fifteen different clusters, three of which comprise less than four DEGs (Fig. 4, Supplementary File 3). DEGs unique to 3Cvs3L comparison constitute the largest cluster with 283 downregulated and 275 upregulated DEGs; both enriched in ADP binding acivity and respectively in defence response with disease resistance protein and inorganic diphosphatase activity.

For shared DEGs between 0v3C and 0v3L, a total of 235 downregulated DEGs (cluster 2) formed the third-largest clusters with the overrepresentation of HSPs, cellular respiration, protein regulation, and response to heat, high light intensity, and hydrogen peroxide. In contrast, the 96 shared upregulated DEGs between 0v3C and 0v3L (cluster 7) were enriched in cell wall biogenesis and carbon utilisation with phospholipase C related to glycerophospholipid metabolism, and carbonic anhydrase involved in nitrogen metabolism. These DEGs represent the common biological processes regulated between D0 and D3 upon pitcher opening.

Clusters 10 (204), 11 (139), 12 (99), and 13 (215) comprise DEGs from fluid protein depletion treatment. The treatment led to the upregulation of unigenes enriched in chromatin regulation, intracellular transport, and defence response with MYB-related LHY TFs. Conversely, unigenes related to energy metabolism, protein regulation, photosynthesis, and cysteine-type endopeptidase activity (cathepsin B) were downregulated.

Clusters 6 (180) and 9 (195) constitute DEGs normally upregulated between D0 and D3 (0v3C) but not after D0 protein depletion, which were enriched in RNA regulation, secondary metabolism, translation, biosynthesis of lignin, secondary cell wall, suberin, pectin and xylan, MYB TF, and a methylenetetrahydrofolate reductase that can metabolise the methyl group of 5,10-methylenetetrahydrofolate to serine, sugars, and starch^39^. Meanwhile, clusters 1 (69) and 5 (68) comprise DEGs normally downregulated in D3 pitcher but not after D0 protein depletion. These DEGs were functionally overrepresented by protein regulation, signal transduction, secondary metabolism, carbohydrate metabolism, urea and water transport, sterol biosynthesis, and pseudo ARR-B TF.

On the other hand, we also performed enrichment analysis of DEGs based on informed functional categorisation as described above (Fig. S5) and hierarchical cluster analysis of DEGs in six main clusters (Fig. S6). All these results corroborate the Venn analysis (Fig. 4) on the transcriptome-wide effect of endogenous fluid protein on pitcher molecular physiology. This led us to speculate that protein depletion resulted in the shift of pitcher physiology away from energy metabolism and photosynthesis, towards secondary metabolism and the replenishment of secreted proteins.

### KEGG pathway analysis

To examine the transcriptional effect of protein depletion treatment on metabolic pathways, we performed KEGG pathway mapping based on KO from KAAS annotation (Supplementary File 4). Fisher's exact test was conducted to identify KEGG pathway enrichment according to 824 KO-annotated DEGs in which 272 of 403 unique KO entries can be mapped to 418 pathways with a background of 2,946 total mapped entries (Table 2).

**Table 2:** KEGG pathway enrichment analysis of DEGs from three pairwise comparisons. Number in parentheses show the total number of mapped KO entry. Up, upregulated; dn, downregulated. Values in the table are enrichment factors based on total mapped KO entry as background. Fisher's exact test with Benjamini-Hochberg multiple test correction: *FDR < 0.05, **FDR < 0.01, ***FDR < 0.001. The list is sorted in the descending order of DEG enrichment factor.

Pathway ID	KEGG pathway	No. mapped KO (2,946)	No. DEG KO (272)	%	DEG	0v3C		0v3L		3CvL	
					(272)	up (99)	dn (68)	up (89)	dn (111)	up (100)	dn (115)
map00940	Phenylpropanoid biosynthesis	22	11	50.0	5.4***	10.8***	9.8**	6.0*	2.4	8.0***	7.0**
map00073	Cutin, suberine and wax biosynthesis	11	5	45.5	4.9***	10.8**	3.9	3.0	0	0	0
map00909	Sesquiterpenoid and triterpenoid biosynthesis	9	4	44.4	4.8**	6.6	4.8	7.4	0	3.3	5.7
map00980	Metabolism of xenobiotics by cytochrome P450	14	4	28.6	3.1**	4.3	3.1	4.7	5.7*	6.3*	1.8
map00053	Ascorbate and aldarate metabolism	18	5	27.8	3.0*	3.3	2.4	1.8	5.9*	1.6	1.4
map04626	Plant-pathogen interaction	33	9	27.3	3.0**	2.7	3.9	5.0*	3.2*	3.6	2.3
map00190	Oxidative phosphorylation	106	25	23.6	2.6*	1.1	0	0.3	5.3***	0.8	2.7**
map00195	Photosynthesis	47	11	23.4	2.5*	1.3	1.8	2.1	5.1***	1.9	2.2
map03010	Ribosome	131	24	18.3	2.0**	2.5**	1.3	1.8	2.2*	1.8	2.5**
map01110	Biosynthesis of secondary metabolites	461	54	11.7	1.3**	1.5*	1.0	1.5	0.6	1.6*	1.4
map01100	Metabolic pathways	1104	115	10.4	1.1*	1.1	1.0	1.0	1.3*	1.1	1.0

Consistent with the Venn analysis (Fig. 4), oxidative phosphorylation and photosynthesis were highly enriched in 0v3L downregulated DEGs, apart from metabolic pathways, oxidative stress-related ascorbate and aldarate metabolism, and metabolism of xenobiotics by cytochrome P450. The enrichment of ribosome in 0v3L downregulated DEGs was counterintuitive based on the expectation that protein replenishment requires increased protein translation. In addition to the suppression of energy metabolism and photosynthesis, the translational mechanism might be downregulated to compensate for a high energy cost of transportation and *de novo* synthesis of secretory hydrolytic enzymes.

Under normal condition (0v3C), the translational mechanism was upregulated together with cutin, suberine and wax biosynthesis. The biosynthesis of secondary metabolites was also highly regulated, especially phenylpropanoids, sesquiterpenoids, and triterpenoids, in which protein depletion treatment resulted in a sustained expression of unigenes which were otherwise downregulated under normal condition (D3C). This might be a compensation for the loss of proteins involved in stress responses, which were highly expressed in newly opened pitchers (D0). Similarly, the enrichment of plant-pathogen interaction in 0v3L DEGs was corroborated by the upregulation of disease resistance proteins (Fig. S6).

### Transcriptional regulation of pitcher fluid proteins and transporters

**Figure 5:**
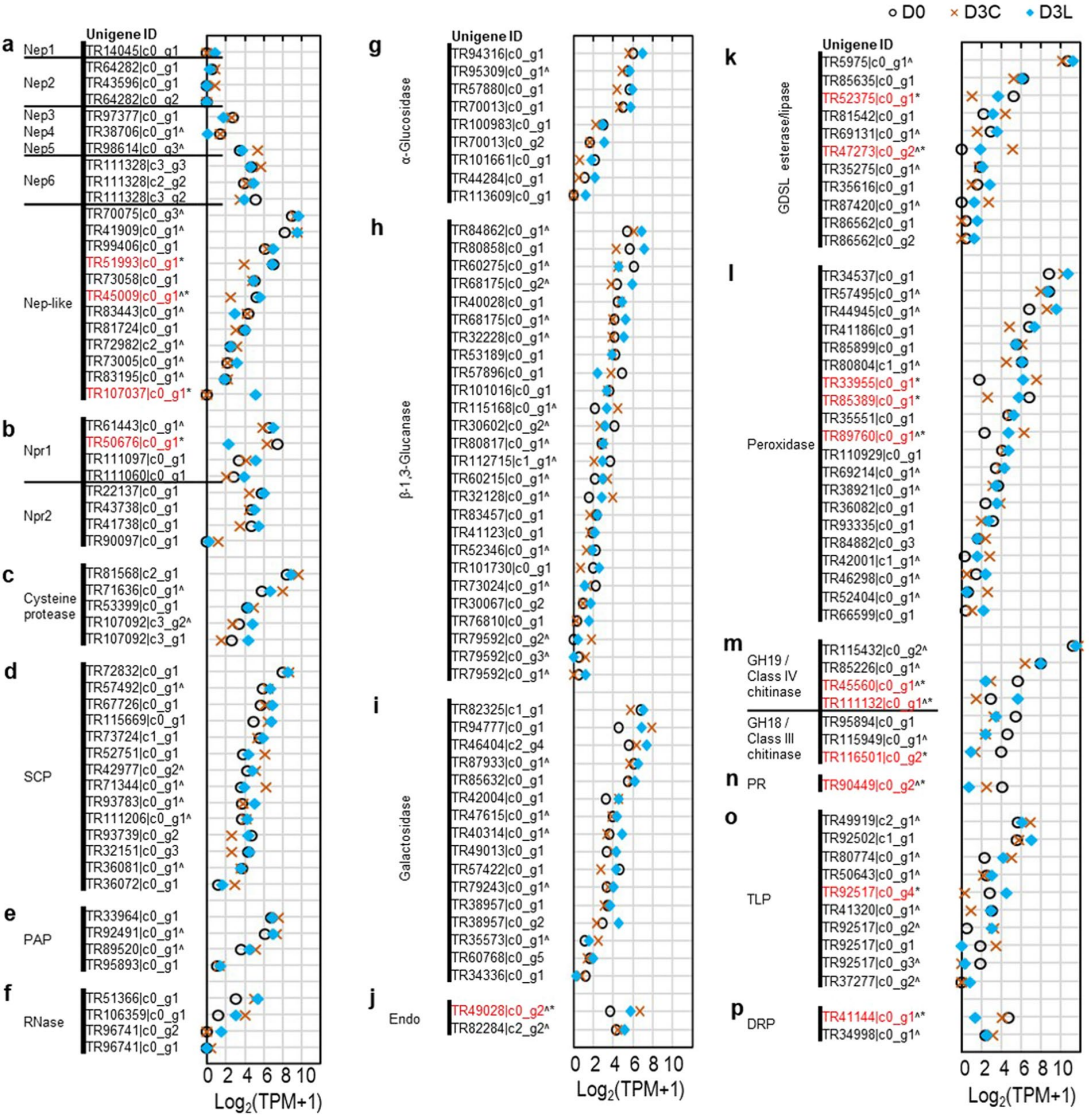
Secretome expression analysis. (**a**) Aspartic protease (**b**) Neprosin (**c**) Cysteine protease (**d**) Serine carboxypeptidase (**e**) Purple acid phosphatase (**f**) S-like ribonuclease (**g**) α-Glucosidase (**h**) β-1,3-Glucanase (**i**) Galactosidase (**j**) Endonuclease (**k**) GDSL esterase/lipase (**l**) Peroxidase (**m**) Chitinase (**n**) Pathogenesis-related protein (**o**) Thaumatin-like protein (**p**) Desiccation-related protein. Asterisk with red font denotes DEG while ^ indicates unigene sequence with the presence of signal peptide from SignalP prediction.

To investigate the effect of endogenous protein depletion on the regulation of protein secretion, we profiled the known secreted proteins and transporters. Based on previous reports on pitcher fluid protein profiling^2,15,16,37^, we identified the homologous unigenes encoding secreted proteins, namely aspartic protease (Nep), neprosin (Npr), cysteine protease, serine carboxypeptidase (SCP), purple acid phosphatase (PAP), S-like ribonuclease (RNase), α-glucosidase, β-1,3-glucanase, galactosidase, endonuclease (Endo), GDSL esterase/lipase, peroxidase (Prx), chitinase (Chit), pathogenesis-related (PR) protein, thaumatin-like protein (TLP), and desiccation-related protein (DRP) (Fig. 5). The highest number of homologous unigenes was found in the glucanase family but none was DEG. Three unigenes encoding Nepenthesin-like proteins, one GDSL esterase/lipase, one peroxidase, one GH19/class IV chitinase, and one TLP were found to have higher expressions in D3L than D3C, compared with lower expressions of one neprosin-1, another GDSL esterase/lipase, one PR, and one DRP.

**Figure 6:**
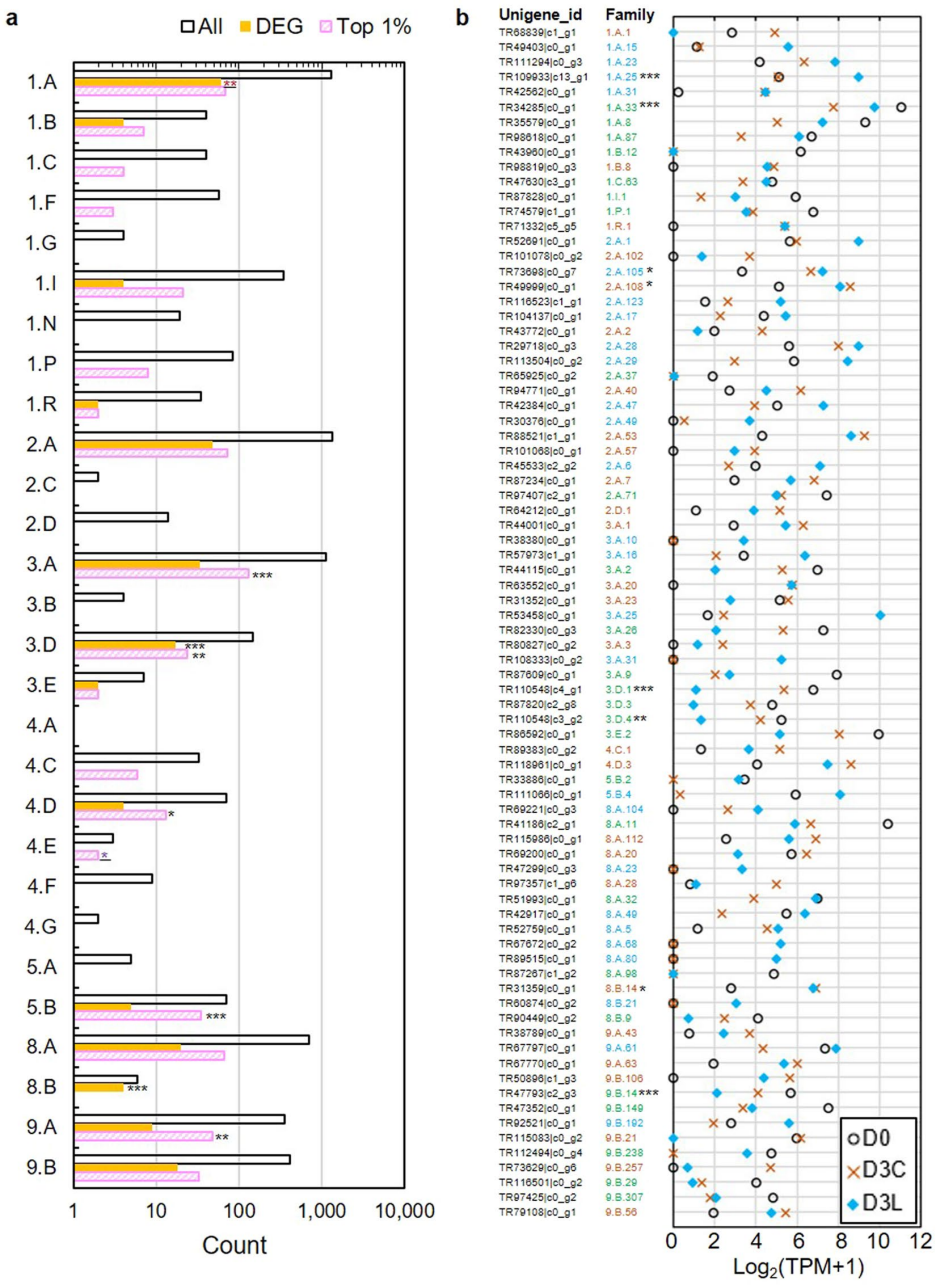
Transportome analysis. (**a**) Enrichment analysis based on TCDB classifications. (**b**) Expression of representative DEGs from each family of transporter based on the highest mean TPM value. The number of DEGs and the top 1% abundant unigenes are shown on a logarithmic scale. Fisher's exact test with Benjamini-Hochberg multiple test correction for overrepresentation analysis: *FDR < 0.05, **FDR < 0.01, **FDR < 0.001. Coloured asterisks represent significance at a specific time point or comparison. Refer Supplementary File 5 for details on TCDB classifications and families.

For transportome analysis based on TCDB annotation (Supplementary File 5), DEGs were highly enriched (FDR < 0.001) with oxidoreduction-driven transporters (3.D) and ribosomally synthesised protein/peptide toxins/agonists that target channels and carriers (8.B) such as pathogenesis-related proteins. The top 1% abundant unigenes were enriched with P-P-bond-hydrolysis-driven transporters (3.A), including ATPases, ABC transporters, and ubiquitin-conjugating enzymes, as well as transmembrane 1-electron transfer carriers (5.B), mainly related to photosynthesis (Fig. 6a). According to transporter family classification (Fig. 6b), defence-related disease resistance protein (1.A.25) and cation channel-forming heat shock protein Hsp70 (1.A.33) were enriched in DEGs with higher D3L expression compared to lower D3L expression of transporters related to respiration and photosynthesis, namely H^+^ or Na^+^-translocating NADH dehydrogenase (3.D.1), proton-translocating cytochrome oxidase (COX) (3.D.4), and putative heme handling protein (9.B.14). On the other hand, mitochondrial pyruvate carrier (MPC) for energy metabolism (2.A.105), laccase for lignin degradation (2.A.108), and metalloendoproteinase-1 for extracellular matrix remodelling (8.B.14) were overrepresented in commonly upregulated DEGs on D3 (Fig. 6b).

Based on transcription factor (TF) analysis (Supplementary File 6), there were only 73 DEGs from a total of 3,469 annotated TFs, in which 12 out of 78 TFs were among the top 1% abundant unigenes (Fig. S7a). MYB TF family involved in plant-specific processes such as development, metabolism, and stress responses^40^ had the highest number of DEGs with seven upregulated in 0v3C but only one in 0v3L (Fig. S7b). Other stress-related alfin-like and HSF TF families were enriched in 0v3L upregulation and downregulation, respectively.

### RT-qPCR validation of RNA-seq analysis

**Figure 7:**
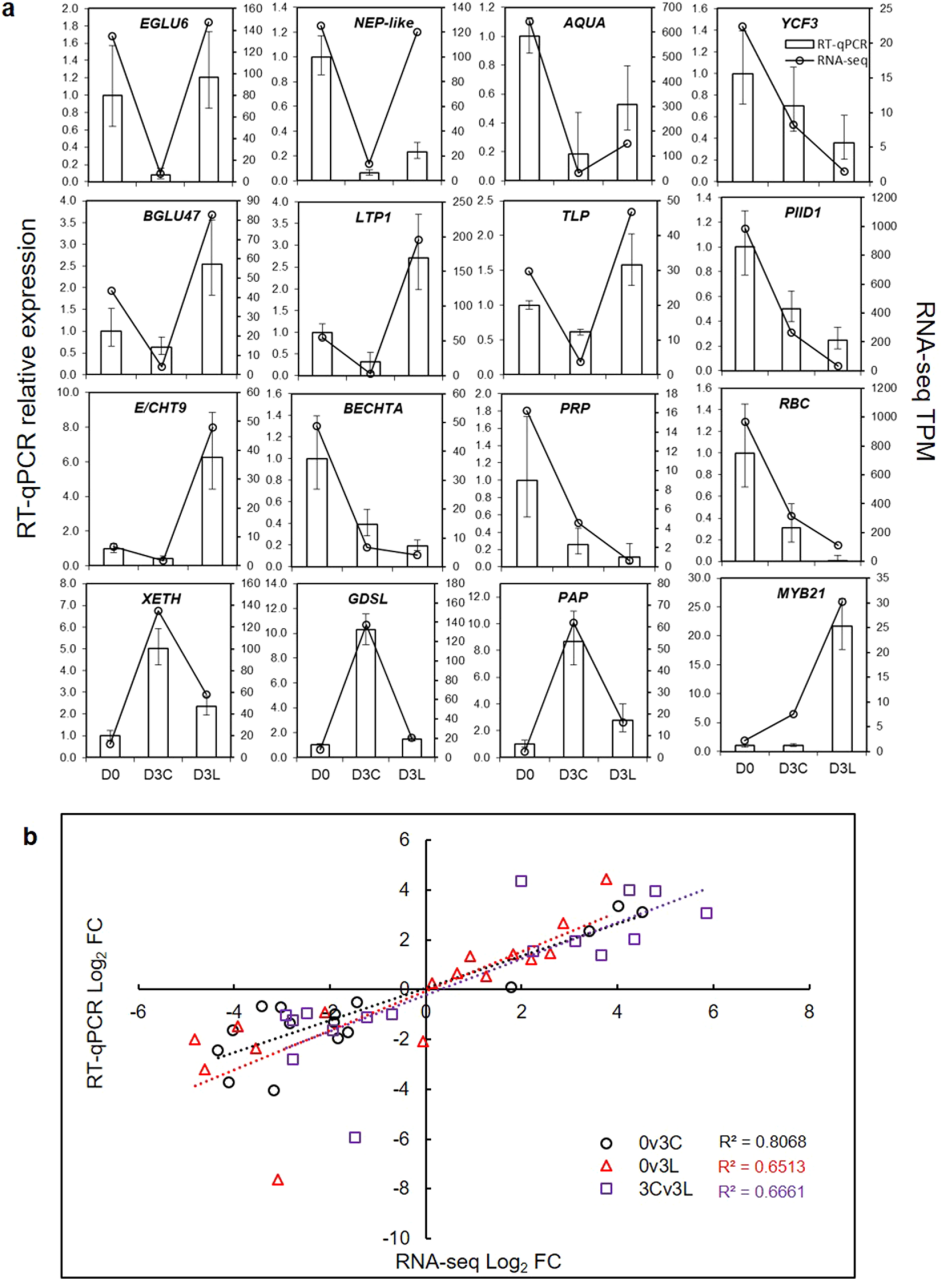
RT-qPCR analysis. (**a**) RT-qPCR analysis of selected genes in comparison to RNA-seq analysis based on relative expression with D0 as the baseline. Error bars represent standard errors from three biological replicates. (**b**) Correlation analysis between RT-qPCR and RNA-seq expression fold-change (Log_2_ FC) for different pairwise comparisons. The coefficient of determination (R^2^) values for different comparisons are shown.

To validate RNA-seq analysis, we performed RT-qPCR analysis using independent samples in triplicates based on a selection of 16 DEGs representative of different aspects of pitcher physiology (Fig. 7). Genes related to photosynthesis, namely *YCF3* (photosystem I assembly protein), *PIID1* (photosystem II protein D1), and *RBC* (RuBisCO) consistently showed lower expression in D3 samples with further downregulation for D3L. A  similar pattern of expression was observed for *PRP* (pathogenesis-related protein) and *BECHTA* (basic endochitinase A). A *NEP-like* (Nepenthesin-like) gene was downregulated o n D3 but to a lesser extent for D3L. Conversely, *EGLU6* (endoglucanase 6) and *AQUA* (aquaporin TIP1-1) showed downregulation in D3C but not D3L. Genes that showed upregulation because of protein depletion in D3L include *BGLU47* (β-glucosidase 47), *LTP1* (lipid transfer protein 1), *TLP* (thaumatin-like protein), *E/CHT9* (Chit9/endochitinase), and *MYB21* (transcription factor MYB21). Meanwhile, *XETH* (xyloglucan endotransglucosylase/hydrolase 30), *GDSL* (GDSL esterase/lipase), and *PAP* (purple acid phosphatase) were upregulated in D3C but not D3L.

The correlation analysis between RT-qPCR and RNA-seq data showed a greater consistency for 0v3C (R^2^ = 0.81) than 0v3L (R^2^ = 0.65) and 3Cv3L (R^2^ = 0.67) (Fig. 7b). This suggests a higher variability for protein depletion samples (3L) compared to control samples (3C). Nevertheless, this indicates good reproducibility of RNA-seq analysis with independent samples used in the RT-qPCR analysis.

## Discussion

The carnivorous plants are a non-model group of plants and there is no genome information available for *Nepenthes* species. Therefore, the transcriptomic analysis provides an alternative to studying the functional genomics of tropical pitcher plants. This reference transcriptome of *N. ampullaria* pitchers based on the previous RNA-seq experiment^38^ is by far one of the most comprehensive reports comparable to *N*. *mirabilis*^16^, *N*. *rafflesiana*^15^, *N*. x *ventrata*^37,41^, and *N*. *khasiana*^6^. Only 5.3% single-copy orthologs were missing based on BUSCO analysis and 31.4% of 158,756 unigenes were functionally annotated, in which 76% were annotated to plants compared to 62% for *N. rafflesiana*^15^. This enabled us to take the next step of research from plant physiology to molecular biology in providing the first glimpse on the transcriptomic composition of young *N. ampullaria* pitchers and transcriptional changes upon endogenous protein depletion.

### Transcriptomic regulation during early pitcher opening

The gene expression changes between D0 and D3 pitchers reflect the transcriptional regulation upon pitcher opening, especially 0v3C DEGs. Meanwhile, the 335 DEGs between 0v3C and 0v3L represent the commonly regulated unigenes not perturbed by protein depletion treatment during young pitcher maturation after lid opening, in which more DEGs were downregulated (238) than upregulated (97) (Fig. 3b). Downregulated DEGs were functionally enriched in cellular respiration, protein regulation, and stress/defence responses (HSPs/disease resistance protein), compared with cell wall biogenesis and carbon utilisation through glycerolphospholipid (phospholipase C) and nitrogen (carbonic anhydrase) metabolisms for upregulated DEGs on D3 (Fig. 4).

Furthermore, ribosome, secondary metabolism, and cutin/suberin/wax biosynthesis were enriched in 0v3C_up DEGs (Fig. S5, Table 2, Supplementary File 3). A functionally diverse MYB TF family was also overrepresented (Supplementary File 6), including MYB39 (TR100930|c0_g1, TR54525|c1_g1) and MYB86 (TR107106|c0_g1) involved in cell differentiation, MYB44 (TR74218|c0_g1) related to hormonal responses^42^, and MYB46 (TR77241|c0_g1) involved in the regulation of secondary wall biosynthesis^43^. This indicates that young pitchers are still developing after pitcher opening with a physiological shift towards secondary metabolism and wax deposition. This is interesting because the waxy zone is absent in *N. ampullaria* pitchers but still retain the slippery peristome^33^. Therefore, it is indicative that peristome development is activated upon pitcher opening. Meanwhile, secondary metabolism involving phenylpropanoids and terpenoids might confer a protective effect against oxidative stress from cellular metabolism^44^. Many secondary metabolites also have antifeedant and antifungal functions, especially flavonoids and naphthoquinones^45^.

On the other hand, most of the unigenes encoding proteins secreted into the pitcher fluids (Fig. 5) already showed high expression levels on D0. This corroborates findings that most of the endogenous secreted proteins already present in pitcher fluids before opening with certain proteins continuously replenished or induced by prey^15,34,37,46^. In comparison, there was no clear trend in the expression of transporters on D0 (Fig. 6). This suggests a more varied regulation of transporters with different functions that might be influenced by different developmental stages or environmental factors. For example, prey induced pitcher fluid acidification via the upregulation of a plasma membrane H^+^-ATPase^47^. Indeed, H^+^ ion fluxes are actively controlled in opened pitchers of *N. ampullaria* to maintain the commensals in less acidic pitcher fluids^28,30^. Four highly expressed aquaporins (1.A.8) for water transport (TR35579|c0_g1, TR58374|c0_g2, TR64049|c0_g1, and TR45806|c0_g1) were downregulated in D3C pitchers (Fig. 6, Fig. 7, Supplementary File 5), which might indicate reduced fluid secretion.

### Pitcher response to endogenous protein depletion

Previous studies focused on the effects of feeding or nutrient supplements in enzyme secretion or cost/benefit analysis of botanical carnivory in relation to assimilatory organ development and physiology^5,32,36,48,49^. None have investigated the effect of protein loss in the trapping organ apart from a recent report of *N*. x *ventrata* pitcher fluid profiling^37^, which is limited by a lack of control samples after 3 days of pitcher opening to discern the effect of pitcher longevity from protein depletion. The study shows that pitchers can respond to the loss of endogenous proteins by continuous secretion of certain proteins, such as aspartic proteinases, neprosin, a serine carboxypeptidase, a β-1,3-glucanase, a PR protein, and a TLP^37^. This is consistent with increased fluid protein content in newly opened *N. alata* pitchers^50^. However, the overall molecular response of pitchers was not discussed.

According to our hypothesis, the protein depletion experiment should trigger the increased expression of certain secreted proteins for the replenishment of proteins greater than 10 kDa or some polysaccharides and secondary metabolites. However, the majority of the proteins found in the secretome analysis were not DEG. Only a class IV chitinase (E/CHT9, TR111132|c0_g1) and a thaumatin-like protein (TLP, TR92517|c0_g4) from the secretome analysis showed such response (Fig. 5). It is surprising to find a prey-induced chitinase gene^46^ to be upregulated by protein loss. Chitinases are antifungal and degrade insect cuticle, in which class IV endochitinases hydrolyse shorter chitin chains derived from the products of class III endochitinases from longer chitin polymers^16,51^. TLP is also antipathogenic and may be involved in carbohydrate metabolism with glucanase activity^52^ and osmotic function in cell wall degradation^53^.

Another TLP (TR107937|c0_g1), a beta-glucosidase (BGLU47, TR42917|c0_g1), and a lipid transfer protein (LTP1, TR42653|c0_g1) not found in the secretome analysis were upregulated in D3L (Fig. 7). Glucanases/glucosidases in carnivorous plants are mostly considered as defences against microbial pathogens because insects contain no glucans^50,54^. Yet, *Drosera rotundifolia* secretes a beta-1,3-glucanase which cleaves plant glucans to simple sugars for absorption^55^. Glucanase could be repurposed in *N. ampullaria* to utilise nutrients from leaf litters. These enzymes may also contribute to the polysaccharidic network in the viscoelastic pitcher fluids for prey capture and retention^13^. LTP could control cuticle permeability and form a cutin barrier to prevent pathogen entry^56^.

The expressions of certain unigenes normally downregulated in D3C were sustained near the D0 levels could also be a response to protein depletion. These include an endoglucanase (EGLU6, TR38761|c0_g1), Nepenthesin-like proteins (TR51993|c0_g1, TR45009|c0_g1), a GDSL esterase/lipase (TR52375|c0_g1), and a peroxidase (TR85389|c0_g1). Peroxidase generates reactive oxygen species (ROS) which releases free radicals and accelerates digestion^57^. Meanwhile, a neprosin (Npr1, TR50676|c0_g1), a purple acid phosphatase (PAP, TR104290|c0_g1), a GDSL esterase/lipase (TR90496|c0_g1), another class IV chitinase (BECHTA, TR45560|c0_g1), a PR protein (TR90449|c0_g2), and a DRP (TR41144|c0_g1) were downregulated in D3L (Fig. 5, Fig. 7).

Taken together, this study indicates that the transcription of some common hydrolytic enzymes found in the pitcher fluids is regulated in response to endogenous protein loss. The pitchers can detect enzymes in the pitcher fluids and respond accordingly to maintain an optimal cocktail of enzymes so that the benefits from prey digestion outweigh the costs of protein replenishment and pitcher metabolisms. Other studies also revealed that pitchers can regulate enzyme activities^24,46,58^. This is likely involving Ca^2+^ and jasmonic acid (JA) signal transduction pathways^22^. In *Drosera rotundifolia*, both chitin and protein applications enhanced chitinase and protease activities^59^. Contrasting chitin, proteins seem to be much better inductors of various enzymes, such as phosphatases, proteases, and chitinases, in pitchers of *Nepenthes*^46^ and *Sarracenia*^60^. Proteins mimic the insect prey best and activate the highest expression of cysteine protease and type I chitinase in Venus flytrap^61^. However, it remains elusive on how the pitchers distinguish the different nature of endogenous enzymes from prey-derived proteins.

Transporters for ammonium, amino acids, and peptides have been found in different pitcher tissues^62^. The expression of cation (ammonium, potassium, and sodium) transporters are shown to be induced in Venus flytrap and JA-regulated^23^. However, none of these transporters was influenced by endogenous protein depletion with only one nitrate transporter (TR79180|c1_g1) in the plasma membrane found to be upregulated and one vacuolar two-pore potassium channel (TR45186|c0_g1) downregulated in D3L. Instead, a few transporters for amines (vacuolar carnitine transporter, TR51977|c0_g1) and carbohydrates (polyol transporter, TR52691|c0_g1 and sugar transporter, TR116523|c1_g1) were upregulated while a nucleobase-ascorbate transporter (TR85860|c0_g2) and two folate-biopterin transporters (TR97407|c2_g1 and TR48965|c0_g1) were downregulated in D3L (Supplementary File 5).

### Trade-offs of protein replenishment

The production of trapping organs by carnivorous plants is a relatively large investment with consequential lower rates of photosynthesis per unit leaf mass and whole plant growth^63^. Interestingly, low photosynthetic rate and stomatal density in *Nepenthes* pitchers were not observed in other unrelated pitcher plants of *Sarracenia*, *Darlingtonia*^48,64^ or *Cephalotus*^65^. The benefits vary with environmental conditions with different trade-offs between energy allocation to carnivory (trap organ, sugar-rich nectar, aroma chemicals, narcotic alkaloids, slippery wax crystals, hydrolytic enzymes, and other biochemicals/volatiles for prey attraction, capture, and digestion) relative to plant competitiveness and fitness (nutrient, photosynthetic rate, and growth)^35^. Unless the costs of making new carnivory organs are less than fluid protein replenishment, the plant is likely to adapt pitcher physiology during stochastic environmental perturbations for maximising yields from the substantial investment in pitcher organs.

Since leaves outperformed pitchers in photosynthesis^1^ and *N. ampullaria* natural habitat is on the forest floor, the shift of pitcher physiology from photosynthesis is a good strategy to further reduce energetic costs. *N. ampullaria* pitchers support a wide range of aquatic fauna, predominantly mosquito larvae^66^, which could decompose leaf litter in addition to bacterial degradation for N sequestration. However, nutrient gain from insectivory is still the best N source compared to detritivory for *N. ampullaria* as an omnivorous plant^1^. Therefore, compared with the complete switch of *N. ampullaria* long-lived pitchers to commensal-dependent detritivory, it is a better investment to maintain prey capture and digestion, such as peristome maturation and replenishment of digestive enzymes, for better nutrient gain from carnivory.

On the other hand, the benefits of rainfall and high humidity in reducing the amount of pitcher fluid secretion could potentially outweigh the chances of spillover or enzyme dilution. This distinguishes wide-mouthed pitchers of *N. ampullaria* with wettable peristomes from narrow-mouthed pitchers with waxy scales of other *Nepenthes* species between rainier and drier areas. Hot and dry weather would increase the costs of maintaining pitcher fluid, whereby emptied pitchers experience much greater stresses. This could explain our personal observations that pitchers are likely to senesce in total loss of proteins, compounds, minerals, and fluid. Therefore, D3L pitchers were refilled with filtrates to simulate only protein loss in this study. We cannot exclude the possibility that some of the DEGs in D3L might be induced by the stress during temporary emptying of pitchers for protein filtration. However, it will be difficult to tease apart such effects in a protein replenishment experiment done *in situ*. Further studies under a controlled environment will be useful to identify the transcriptional response to pitcher fluid loss to be distinguished from protein loss per se.

The MYB21 TF (TR34238|c0_g1) involved in the regulation of light-induced genes^67^ and response to jasmonate^68^ was upregulated specifically in D3L (Fig. 7, Supplementary File 6). Notably, a sigma70-like TF (TR104595|c0_g2), which controls photosynthetic efficiency through photosystem stoichiometry^69^, was found to be uniquely downregulated in 0v3L. Meanwhile, secondary metabolism particularly phenylpropanoid biosynthesis was activated in D3L (Table 2). Jasmonates (JAs) mediate the production of secondary metabolites involve in plant defences^70,71^. JAs signal the reprogramming of gene expression from photosynthesis and growth with significant costs of resource allocation to defence as shown in the Venus flytrap^23^. The fitness benefits from defence might compensate for the costs^22^. During prey capture, photosynthesis is transiently inhibited while respiration is stimulated, similarly to non-carnivorous plants in response to wounding or stress^72,73^.

Interestingly, the transcriptome-wide response of pitchers upon protein depletion indicates a shift away from both photosynthesis and respiration through the downregulation of gene expression (Table 2). These genes include NADH-ubiquinone oxidoreductase and transporters related to respiration and photosynthesis, namely H^+^ or Na^+^-translocating NADH dehydrogenase, proton-translocating cytochrome oxidase (COX), and putative heme handling protein (Fig. 4, Fig. 6). Adaptive changes in COX have been reported to provide respiratory power in the carnivory evolution of Utricularia bladderwort plants^74^.

In carnivorous plants, JA signalling plays dual functions of defence and the induction of digestive enzymes^22^. In *Sarracenia*, there is no systemic induction of hydrolytic enzymes in other pitchers, suggesting an independent response of each pitcher^60^. Under a nutrient-poor environment, physiological response restricted to only local pitchers will be more favourable to save limited resources. Therefore, we speculate that the response to protein loss, as a stochastic event, is also at an individual pitcher-level. Further study will be required to determine the trade-offs and systemic transcriptional/physiological responses, if exist, in other pitchers at the whole-plant level. It will also be interesting to apply the same approach to investigate local and systemic responses to exogenous protein application or prey capture in the future.

Despite the similarity to JA response, none of the genes involved in the biosynthesis and signalling of JA was found to be differentially regulated in this study (Supplementary File 3). Furthermore, a Tify TF family that represses JA responses^75^ was found to be enriched in the top 1% abundant unigenes unique to D3L (Fig. S3). Hence, the involvement of JA in pitcher response to protein loss will require further investigation.

## Conclusion

One strength of this protein depletion experiment was the use of filtrate to minimise the confounding effects from the loss of ions or metabolites in the pitcher fluids and to prevent pitcher senescence. A great challenge was the on-site monitoring of pitcher development to capture the timing of pitcher opening for performing this experiment in a controlled manner. The same experiment was independently repeated for RT-qPCR validation of gene expression. Therefore, the results obtained from the RNA-seq analysis are reliable and provide valuable data for subsequent in-depth experiments. This study as summarised in Fig. S8 contributes significantly to our knowledge and understanding of molecular responses of carnivory organs during protein loss in pitcher plants. Overall, our study suggests a trade-off between photosynthesis, resource allocation, and organ defence during endogenous protein replenishment in the pitcher fluids.

## Materials and Methods

### Sample acquisition and RNA extraction

*Nepenthes ampullaria* Jack plants originally from Endau-Rompin National Park were grown under a shady environment at Universiti Kebangsaan Malaysia experimental terrace (2°55′09.0”N, 101°47′04.8”E). Developing pitchers were monitored *in situ* and covered using clear drawstring gauze bags to prevent insect entry. For the previously described RNA-seq experiment^38^, samples were collected from Jan 2014 to Apr 2015 in the morning 9 - 10 am, including pitchers within 24 hours of opening as day 0 control (D0) and pitchers after three days since opening (D3C) for longevity experiment. For the endogenous protein depletion experiment, fluids of newly opened pitchers were syringe filtered with 0.22 µm polyvinylidene difluoride (PVDF) membrane before ultrafiltration (10 kDa molecular weight cut-off) with Microsep Advance device (PALL, USA), replaced into respective pitchers on-site within one hour and covered before collection after three days (D3L). Individual whole pitchers excluding tendril were emptied, rinsed with distilled water before flash-frozen in liquid nitrogen and stored at −80 °C prior to extraction. A new independent set of samples was collected in at least triplicates between Aug to Sep 2017 with the same experimental design for RT-qPCR validation. Total RNA was extracted based on a modified CTAB method^76^.

The RNA-seq experiment was described in the previous data article with primary analysis^38^. Briefly, poly-A enriched cDNA libraries were prepared and sequenced using Illumina HiSeq 2500 for 125 bp paired-end reads by Macrogen, South Korea. Adapter sequences and raw reads with Phred score < 25 were removed from raw reads using Trimmomatic^77^. The processe reads were assembled and analysed through the Trinity analysis pipeline (v2.0.6), using default parameters with k-mer size of 25^78^. This Transcriptome Shotgun Assembly project has been deposited at DDBJ/ENA/GenBank under the accession GFAE00000000. The version described in this paper is the first version, GFAE01000000. The unigene fasta file was generated based on the gene-to-isoform relationship using SuperTranscript utility in the Trinity analysis pipeline.

### Functional annotation

The contigs were annotated with the Trinotate pipeline^79^. Translated peptides were generated using TransDecoder for BLASTP and other protein-based analyses in the Trinity pipeline. Trinotate annotation suite provides multiple homology searches, including BLASTX (vers. 2.3.0+), BLASTP (vers. 2.3.0+), HMMSCAN against Pfam, SignalP, TMHMM, and RNAMMER. All results were stored into the Trinotate SQLite database template and a spreadsheet-based summary report was generated from Trinotate using BLAST E-value cut-off of 1E^−5^. Gene Ontology (GO) terms were assigned to unigenes with identified hits against PFAM (vers. 27.0) and Swiss-Prot (ver. 2016_03) databases in the categories of cellular component, biological process, and molecular function. The GO terms were visualised using Web Gene Ontology Annotation Plotting web tool (WEGO 2.0)^80^. BUSCO v3 analysis^81^ was performed using a lineage dataset of embryophyta_odb10 (2017–12–01) comprising 60 plant species with 1,375 BUSCO IDs.

Transcription factor was annotated using standalone software iTAK v1.7 with database v18.10^82^ and combined with Arabidopsis annotation^83^. Local BLASTN was performed against TAIR10 Arabidopsis sequence database (https://www.arabidopsis.org/) with cut-off E-value of 1E^−5^ to identify homologous genes for comparative analysis based on Arabidopsis. The annotation and classification of the transporter were based on the Transporter Classification Database (TCDB) accessed on May 16, 2019^84^.

KEGG Automatic Annotation Server (KAAS) was used to annotate the transcriptome with KEGG Orthology (KO) identifiers or K numbers that are associated with enzymatic functions^85^. BLAST alignment method was performed against the database comprising all available plant species, human, and default species set for eukaryotes and prokaryotes. Single-directional best hit (SBH) was chosen as the assignment method. Unigenes with annotated KO numbers were mapped against KEGG PATHWAY database (May 1, 2019) with KEGG Mapper^86^ and tabulated for enrichment analysis.

### Gene abundance estimation and differentially expressed gene (DEG) analysis

Raw reads for all samples were aligned to the reference transcriptome assembly using Bowtie^87^ as part of Trinity workflow. The estimation of transcript abundance from each sample was performed using RSEM (RNA-Seq by Expectation-Maximization)^88^ by following default settings with the alignment results. Pairwise DEG analysis was performed at the unigene level based on the read counts using the edgeR Bioconductor package^89^ with TMM normalisation. Significant DEGs are defined by stringent parameters of false discovery rate (FDR) < 0.001 and |Log_2_FC|> 2. Unigene abundance is expressed as Transcripts Per Kilobase Million (TPM), which normalised for gene length and read depth for better comparison between samples.

Enrichment analysis of DEGs (Fisher's exact test with Benjamini-Hochberg multiple test correction) was performed using Perseus software v1.6.5.0^90^ based on a cut-off threshold of 0.05. Enriched GO terms were visualised using online tool REViGO (http://revigo.irb.hr/)^91^. Cytoscape software with Biological Network Gene Ontology (BiNGO) plugin^92^ was used to assess DEG overrepresentation of GO categories and visualisation using EnrichmentMap plugin^93^.

### Reverse transcription quantitative PCR (RT-qPCR) analysis

First-strand cDNA was synthesised using 250 ng of total RNA extracted from an independent set of samples in triplicates using Maxima H Minus First Strand cDNA synthesis kit (Thermo Scientific Inc., USA). The cDNA was adjusted to a 12.5 ng/µL concentration and diluted in a 1:10 ratio for standard curve analysis to evaluate primers’ efficiencies. The primers were designed using Primer-BLAST^94^ as listed in Table S1. Reverse transcription quantitative real-time PCR (RT-qPCR) was performed using Eco^TM^ Real-Time PCR System with Eco Study (v5.2.12) software using iTaq^TM^ Universal SYBR^®^ Green Supermix (Bio-Rad). PCR reactions include 1 µL of cDNA template (12.5 ng), 5 µL of master mix (2×), 1 µL of 10 µM of each forward and reverse primer and nuclease-free water to a 10 µL final volume. The amplification programme was as follows: 95 °C for 2 min and 40 cycles of 95 °C for 5 s, and 55 to 60 °C for 30 s. PCR specificity was confirmed by end-cycle melt curve analysis and no-template controls (NTCs). Primer efficiency was determined based on a standard curve with five serial dilutions. RT-qPCR analysis was performed using three biological replicates, each with three technical replicates. Expression of target genes were normalized based on internal reference elongation factor 1 (*EF1A*) (TR100191|c0_g2) and actin (*ACT7*) (TR40722|c2_g12) using 2^-ddCt^ method^95^. Statistical analysis was performed using linear regression to obtain the correlation between relative expression data of qPCR and RNA-seq.

## Supplementary information

Supplementary File 1.

Supplementary File 2.

Supplementary File 3.

Supplementary File 4.

Supplementary File 5.

Supplementary File 6.

Supplementary Tables and Figures.

## Data Availability

All analysis and sequence files can be accessed at 10.6084/m9.figshare.8105189. The transcriptome assembly generated from the SRA reads under the BioProject accession number PRJNA299862 is available through NCBI TSA with the following link: https://www.ncbi.nlm.nih.gov/nuccore/GFAE01000000.
