# Supplementary material for: Transcriptome-wide shift from photosynthesis and energy metabolism upon endogenous fluid protein depletion in young Nepenthes ampullaria pitchers
Source: Sci Rep. 2020 Apr 20;10:6575. doi: 10.1038/s41598-020-63696-z (PMC7170878; doi:10.1038/s41598-020-63696-z)
Supplement: Supplementary file 7 — Supplementary Tables and Figures. [file 41598_2020_63696_MOESM7_ESM.pdf]

Transcriptome-wide shift from photosynthesis and energy metabolism upon endogenous fluid protein depletion in young *Nepenthes ampullaria* pitchers

Hoe-Han Goh, Anis Baharin, Faris 'Imadi Mohd Salleh, Rishiesvari Ravee, Wan Nor Adibah Wan Zakaria, Normah Mohd Noor

Institute of Systems Biology, Universiti Kebangsaan Malaysia, 43600 UKM Bangi, Selangor, Malaysia.

*Correspondence E-mail: gohhh@ukm.edu.my; Tel.: +60-3-8921-4557

Supplementary Information

Table S1. Sequences of primers used in the RT-qPCR validation of RNA-seq analysis.

Unigene ID	Gene	Sequence (5'→3')	Primer Tm (°C)	%GC	Product size (bp)	Primer efficiency (%)
TR100191|c0_g2	*EF1A*	GGAGGTGTGGCAATCGAGAA	60.0	55	103	100.3
Elongation factor-1A		TCCAGCTAAGGAGGCTGCTA	60.0	55		
TR40722|c2_g12	*ACT7*	GGTGCTGAGAGATGCAAGGA	59.8	55	127	97.5
Actin-7		TGCAGACAGGATGAGCAAGG	60.0	55		
TR90496|c0_g1	*GDSL*	TTTGGTTGGGTGCAGTGAGT	60.0	50	180	100.0
GDSL esterase/lipase		ACTCACCTTGCTGCTCCTTC	60.0	55		
TR51993|c0_g1	*NEP-like*	AGCGTTGGGTGAGAAGACTG	60.0	55	148	100.1
Nepenthesin-like		CCTCAACACCGCCAACTACT	60.0	55		
TR90449|c0_g2	*PRP*	AGAGCATAAGCCGCAACAGT	60.0	50	86	103.8
Pathogenesis-related protein		GACTTCCTCGATGCCCACAA	60.0	55		
TR38761|c0_g1	*EGLUC6*	CCAGCTCCTTCCTGTTGAGG	60.0	60	139	107.1
Endoglucanase 6		CCATGAAGTCGTTGCCCTCT	60.0	55		
TR73095|c0_g1	*XETH*	TACTGCTACGACACCTTGCG	60.1	55	93	99.7
Xyloglucan endotransglucosylase/hydrolase		CAGCCTGCCATTCTCCTTGA	60.0	55		
TR42917|c0_g1	*BGLU47*	TGAGTGTGAAGCAGGAGCAG	60.0	55	179	107.7
Beta-glucosidase 47		ACACCGGCACATTTCCGTAT	60.0	50		
TR42653|c0_g1	*LTP1*	GCTTGAAGCTGCTGTTTGCT	60.0	50	128	110.3
Lipid transfer protein-1		AGCAGGTAAACCCAGGAGGA	60.2	55		
TR35579|c0_g1	*AQUA*	TAGCCATCGGGTTCATCGTG	59.9	55	99	98.1
Aquaporin TIP1-1		TAACTAAAGCCGGGCCGAAG	60.1	55		
TR77098|c0_g1	*YCF3*	GTTGCGGTCATGAATGTTGG	58.0	50	111	96.3
Photosystem I assembly protein		AGTGGTTCAAGGCGTAGCAT	59.7	50		
TR86592|c0_g1	*PIID1*	TGGAGGAGCAGCAATGAAGG	60.0	55	149	92.5
Photosystem II Protein D1		GCGAAAGCGAAAGCCTATGG	60.0	55		
TR30058|c0_g1	*RUBISCO*	CAGTCGTTGTCCCATCACCA	60.0	55	180	102.4
RuBisCO large subunit alpha		TCGGTCCCAGAGGGAGAAAT	60.0	55		
TR104290|c0_g1	*PAP*	AGCTCATCGGCAACAATGGA	60.0	50	162	101.8
Purple acid phosphatase		CAGAGAAGGGCTTGCCATGA	60.0	55		
TR111132|c0_g1	*E/CHT9*	ATCGAAATAACGCCGCTTGC	60.0	50	140	103.8
Chit9/Endochitinase		CTGGCCCAAGAAAGCAGCTA	60.3	55		
TR45560|c0_g1	*BECHTA*	CTGCTCCCTCACGAAACAGT	60.0	55	102	102.9
Basic endochitinase A		ATCGCTGCTTTCCTTGGTCA	60.0	50		
TR107937|c0_g1	*TLP*	GACGATGGACCAGGAAAGGG	60.1	60	87	122.4
Thaumatin-like protein		ACCAACAGAACACACGCTCT	59.8	50		
TR34238|c0_g1	*MYB21*	CCACCACAGCCTACTTCCTG	60.0	60	148	113.3
Transcription factor MYB21		GCGCTAGCTTCCATTTCAGC	60.0	55		

Table S2. Distribution of unigenes based on TPM values in different samples. Mean TPM was calculated based on average TPM values from three samples.

TPM	D0		D3C		D3L		Mean TPM	
	Number	%	Number	%	Number	%	Number	%
0	83,084	52.33	86,059	54.21	67,507	42.52	26,146	16.47
>0-1	20,820	13.11	26,609	16.76	44,196	27.84	89,909	56.63
>1	54,852	34.55	46,088	29.03	47,053	29.64	42,701	26.90
>1-10	42,869	27.00	32,333	20.37	32,661	20.57	28,935	18.23
>10-100	11,043	6.96	12,361	7.79	12,953	8.16	12,521	7.89
>100-1000	863	0.54	1,323	0.83	1,367	0.86	1,180	0.74
>1000	77	0.05	71	0.04	72	0.05	65	0.04

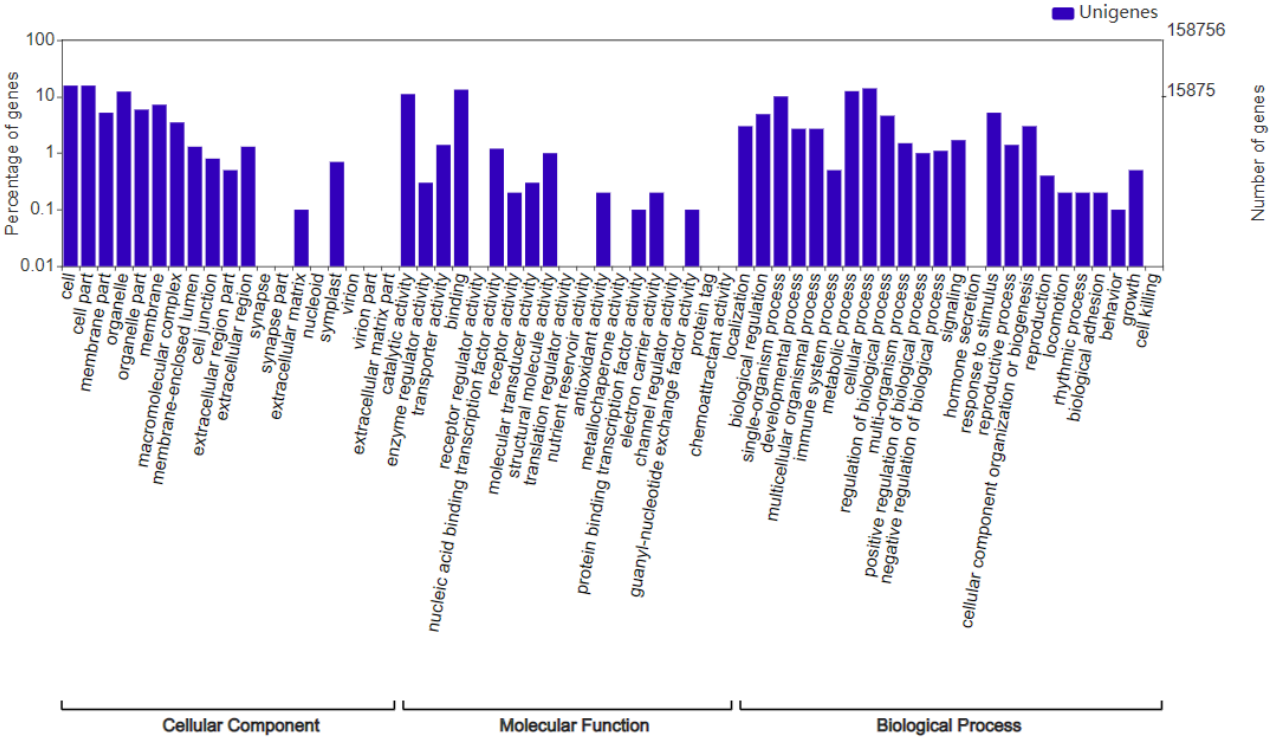

Figure S1. Gene ontology (level 2) term distribution of unigenes based on WEGO analysis.

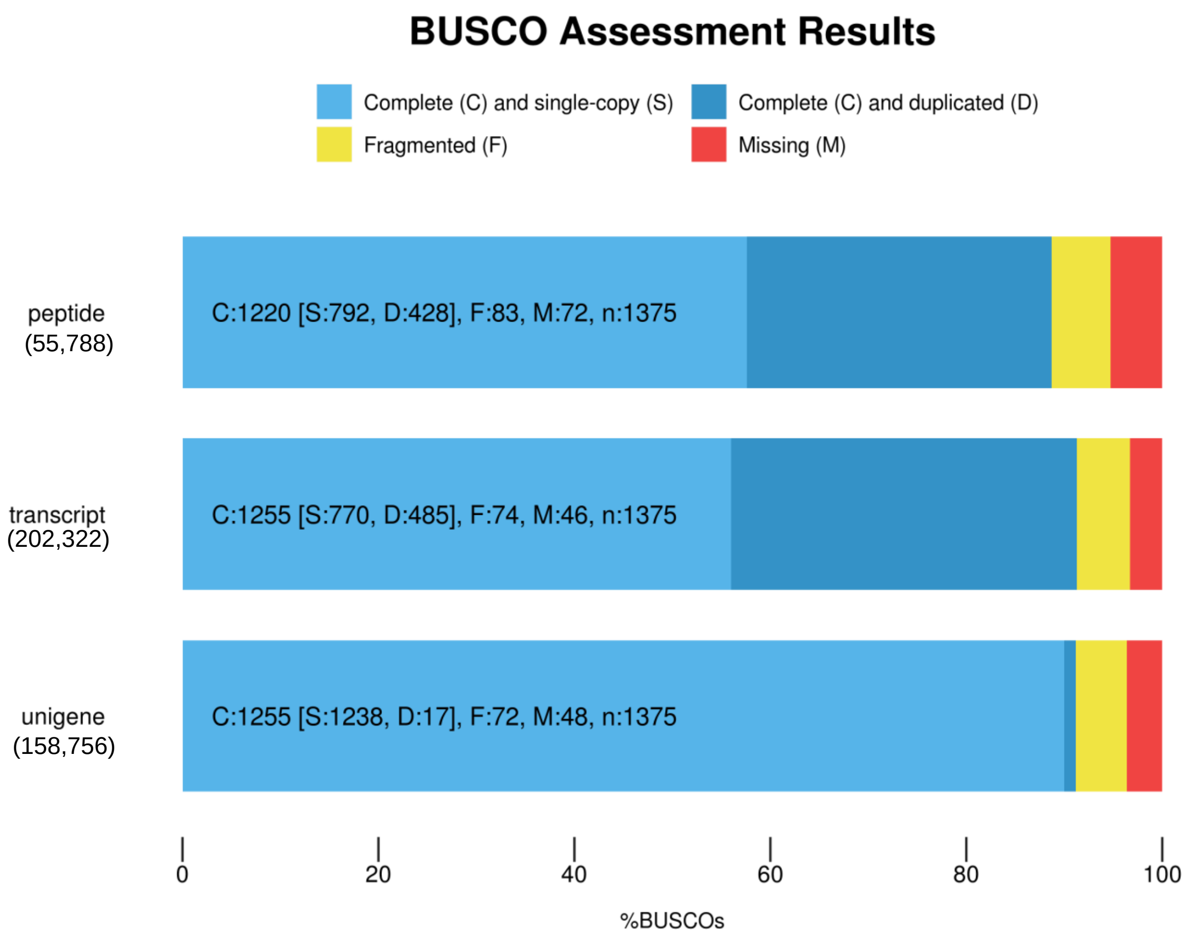

Figure S2. Summary of BUSCOv3 analysis based on predicted peptide and transcript sequences with combined analysis at the unigene level. Lineage dataset: embryophyta_odb10 (Creation date: 2017-12-01, number of species: 60, number of BUSCOs: 1,375). The number of sequences used for annotation is stated in parentheses. Refer Supplementary File 1 for BUSCO annotation.

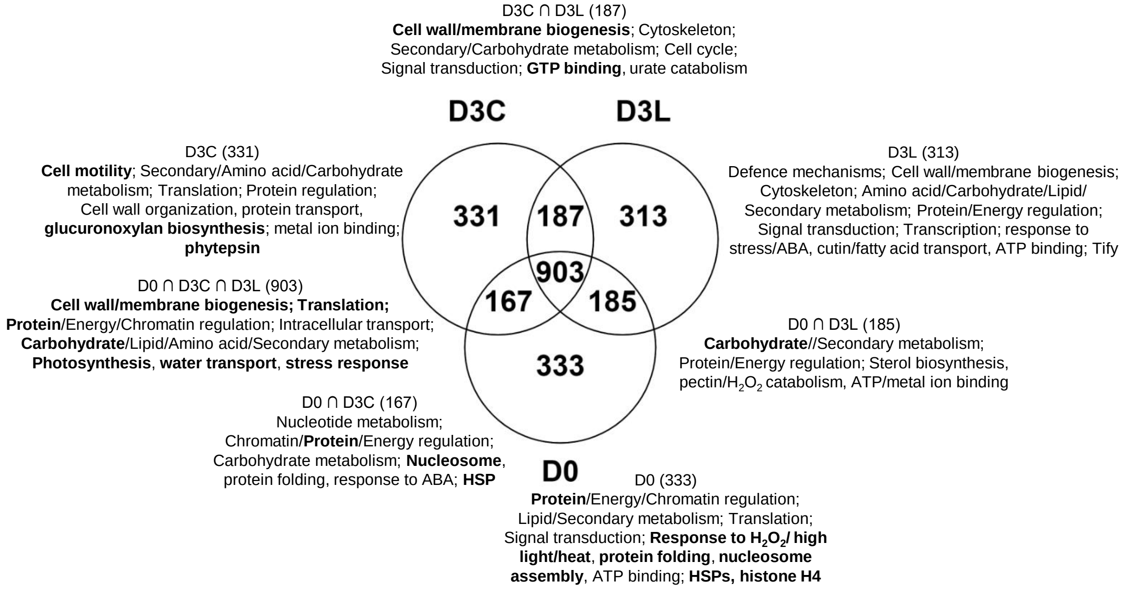

Figure S3. Venn analysis and enrichment analysis of the top 1% (1,588) abundant unigenes from each sample based on functional annotations. Fisher's exact test with Benjamini-Hochberg multiple test correction cut-off values of FDR<0.05. Listed according to the descending order of enrichment factors based on the background of all unigenes. Bolded fonts represent significant enrichment based on stringent background of only annotated unigenes in respective categories, namely GO (34,444), KOG (15,577), and KO (25,512). Refer Supplementary File 2 for details.

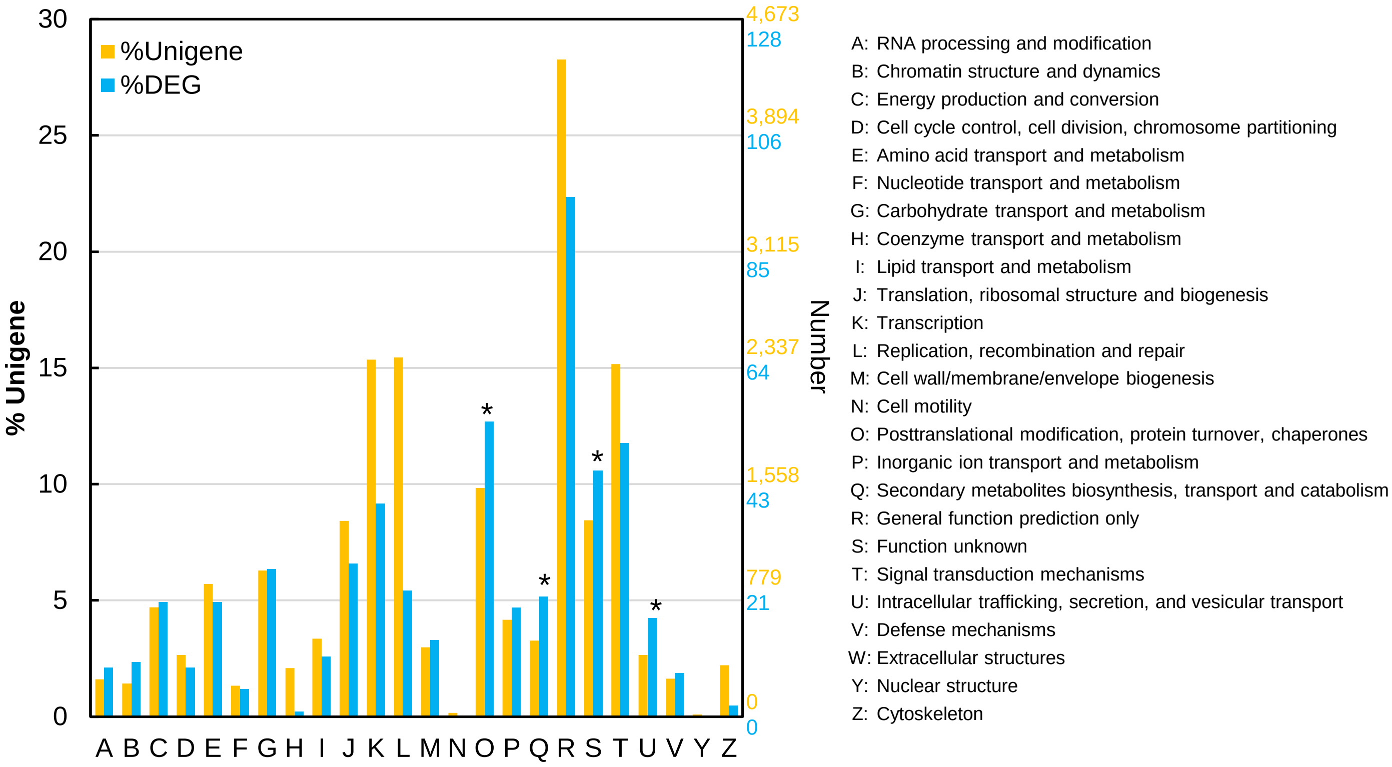

Figure S4. Eukaryotic Orthologous Groups (KOG) classification of transcriptome and the 2,064 DEG based on eggNOGv3 classification. Fisher's exact test with Benjamini-Hochberg multiple test correction: *FDR<0.05, **FDR<0.01, ***FDR<0.001. Background of 15,577 unigenes and 425 DEGs annotated with KOG.

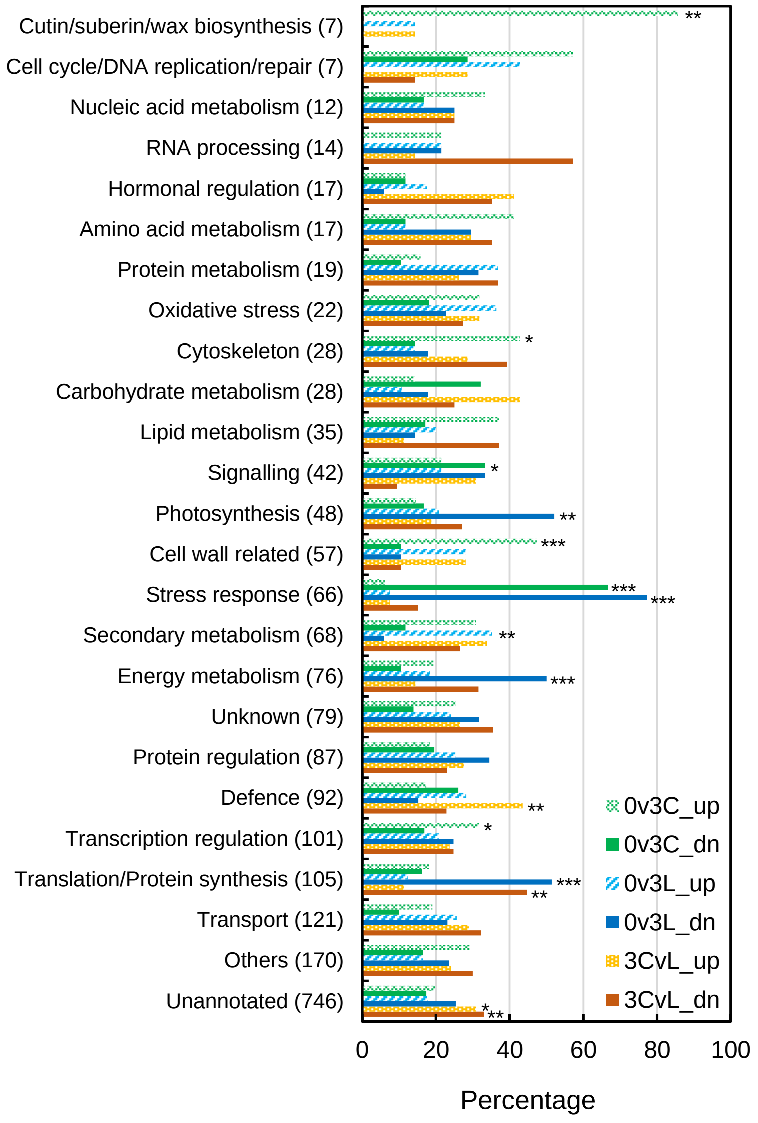

Figure S5. Functional enrichment analysis of 2,064 categorised DEGs. The percentage is calculated based on the proportion of DEGs in each sample against the total number of DEGs in all samples (numbers in parentheses) for the respective categories. Fisher's exact test with Benjamini-Hochberg multiple test correction: * FDR<0.05, ** FDR<0.01, *** FDR<0.001.

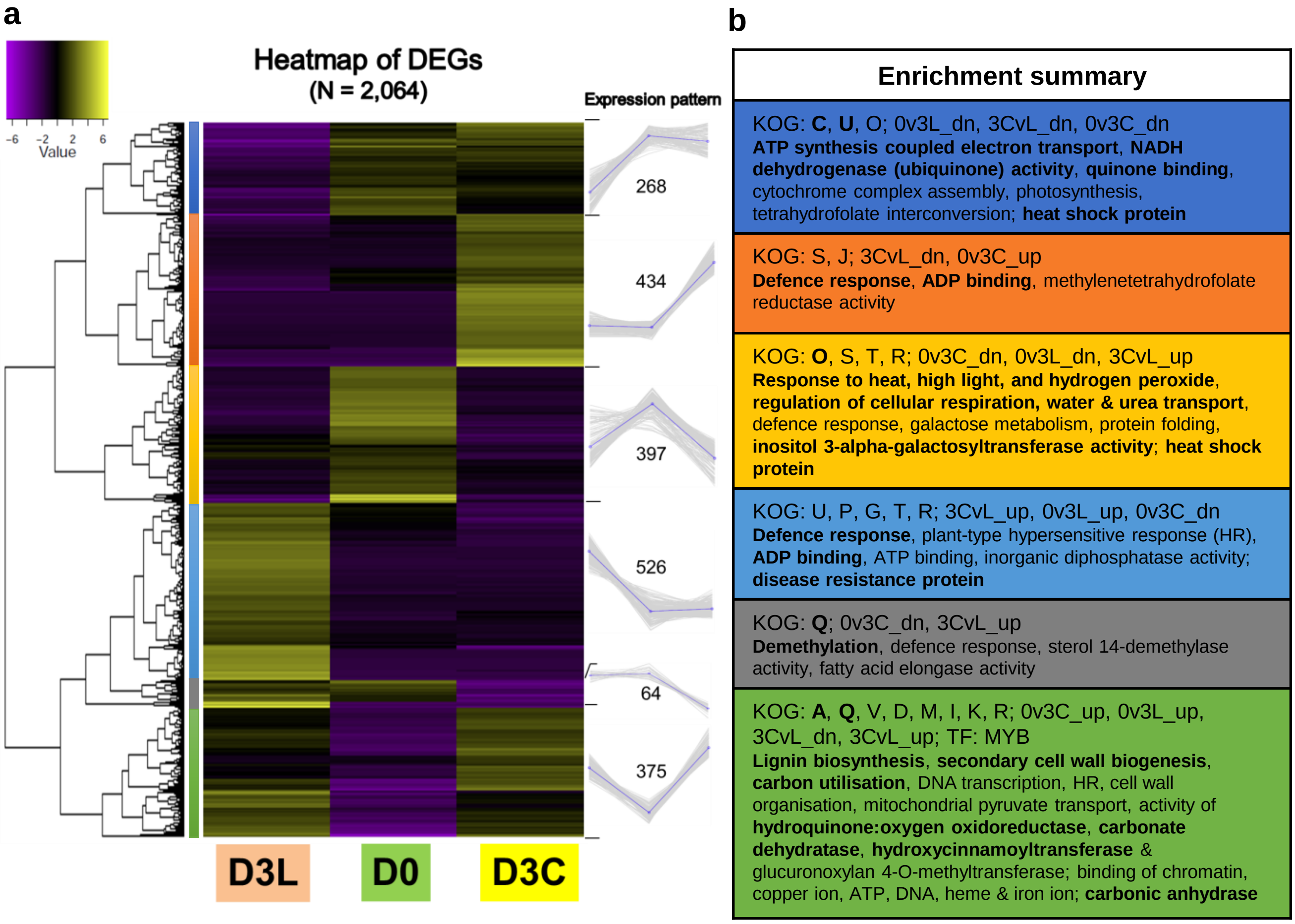

Figure S6. DEG clustering and enrichment analysis. (a) Heatmap with hierarchical clustering is based on median-normalized Fragments Per Kilobase Million (FPKM) values of 2,064 upregulated (up) and downregulated (dn) DEGs from all comparisons. Colour bars correspond to six clusters. Profile plots show the respective number of DEGs with median expression patterns shown as blue lines. D0/0: day 0 control; D3C/3C: day 3 longevity experiment; D3L/3L/L: day 3 endogenous protein depletion experiment. (b) Summary of enrichment analysis based on all 158,756 unigenes as background. Fisher's exact test with Benjamini-Hochberg correction at cut-off corrected *P*-value of 0.05. The list is sorted according to the descending order of enrichment factor/significance. Descriptions based on overrepresented GO terms are not exhaustive. Bolded fonts represent significant enrichment based on a stringent background of only annotate unigenes in respective categories. Refer Supplementary Material 3 for details.

a

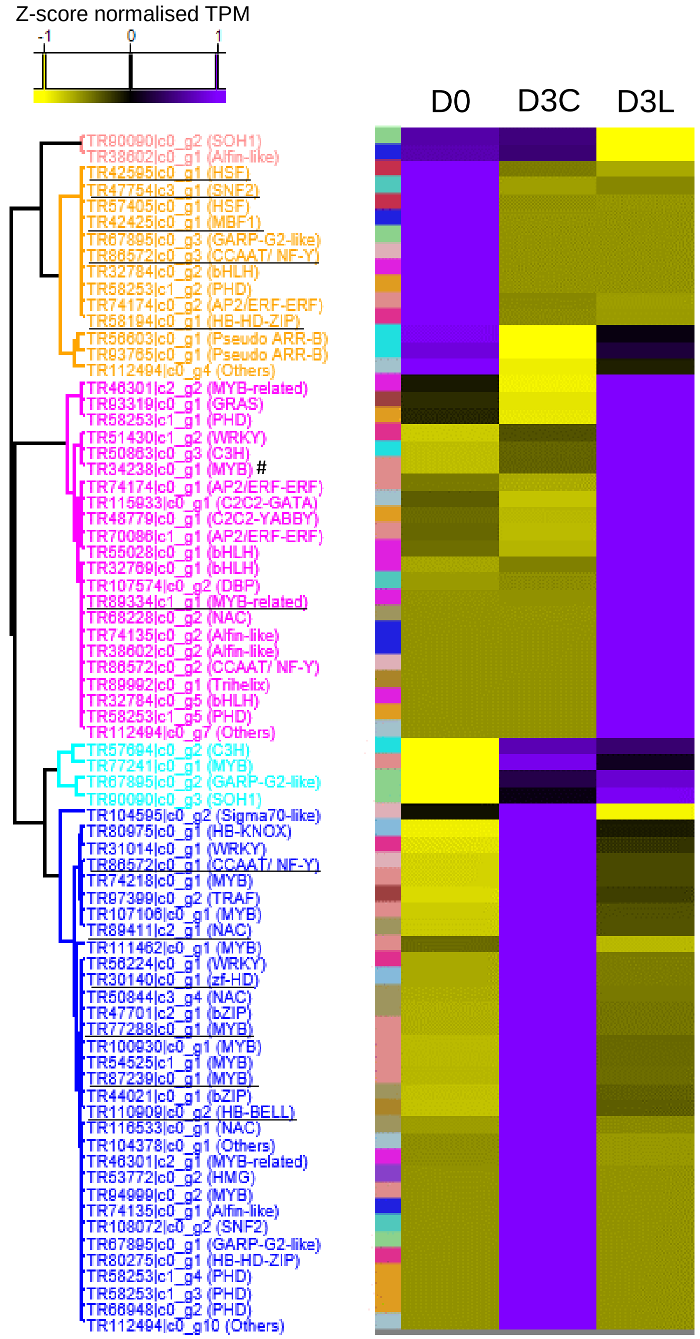

b

TF family	All	DEG	0v3C		0v3L		3CvL	
			up	dn	up	dn	up	dn
MYB	217	10**	7***	0	1	0	0	2
PHD	85	6**	2	1	1	1	2	3**
Alfin-like	31	4**	0	0	2**	1	2**	2**
CCAAT/ NF-Y	66	3	1	1	2*	1	1	0
HSF	37	2	0	1	0	2**	0	0
Pseudo ARR-B	15	2*	0	2***	0	0	0	0
SOH1	4	2**	1*	0	1*	1*	0	1*
MBF1	4	1	0	1*	0	1*	0	0
Sigma70-like	8	1	0	0	0	1*	0	1*

Figure S7. Transcription factor analysis. (a) Hierarchical cluster analysis of all 73 differentially regulated transcription factors. Colour bar according to TF family. Unigene id is coloured according to five clusters based on similarity in expression patterns. Twelve TFs in the top 1% abundant unigenes are underlined. (b) The number of members in each TF family found to be significantly enriched. Fisher's Exact test: *P<0.05, **P<0.01, ***P<0.001. up: upregulated; dn: downregulated. Refer Supplementary Material 6 for details.

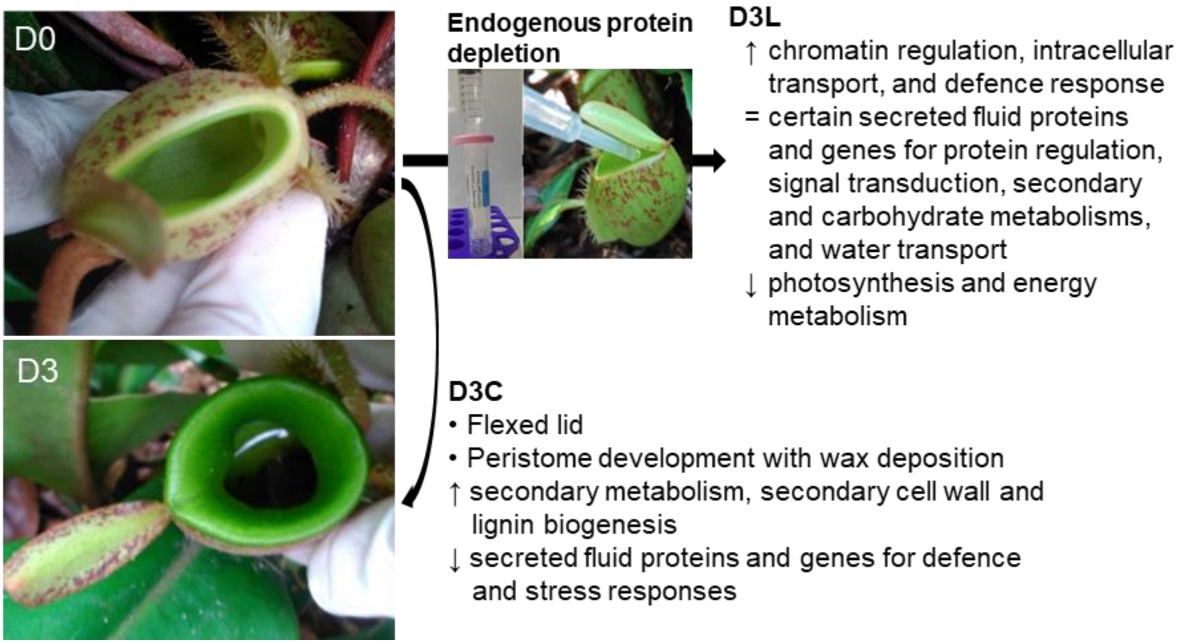

Figure S8. Graphical summary of results.
